# Sociodemographic Correlates of Modifiable Risk Factors for Hypertension in a Rural Local Government Area of Oyo State South West Nigeria

**DOI:** 10.1155/2014/842028

**Published:** 2014-12-21

**Authors:** Saliu Abdulsalam, Adenike Olugbenga-Bello, Olakunle Olarewaju, Ismail Abdus-salam

**Affiliations:** ^1^Department of Community Medicine, Ladoke Akintola University of Technology Teaching Hospital, Ogbomoso, Oyo State, Nigeria; ^2^Department of Community Medicine, Faculty of Clinical Sciences, College of Health Sciences, Ladoke Akintola University of Technology, Osogbo, Nigeria; ^3^Directorate of Disease Control, Lagos State Ministry of Health, Alausa, Ikeja 100282, Lagos State, Nigeria

## Abstract

Modifiable risk factors of hypertension contribute significantly to all-cause morbidity and mortality worldwide. The study aimed to determine the prevalence of and the association of modifiable risk factors with hypertension in rural community. A cross-sectional study was conducted among 166 male and 201 female adults of 18 years and above using cluster sampling technique. Data were collected using modified WHO STEPS instrument and hypertensive subjects were defined as those with systolic greater than or equal to 140 and diastolic of 90 mmHg. Data were analyzed with SPSS version 17 with level of significance at *P* < 0.05. The mean age of the subjects was 36.36 (±16.88) years and mean systolic and diastolic pressures were 124 (±16.93) and 76.32 (±11.85) mmHg, respectively. The prevalence of hypertension was high (22.9%) in this rural communities but awareness was low, 10.71%. The prevalence of alcohol consumption, sedentary lifestyle, abnormal weight, inadequate sleep, smoking, significant stress, and female use of hormonal contraceptives was 149 (40.6%), 91 (24.8%), 88 (24.0%), 122 (33.2%), 14 (3.8%), 65 (17.7%), and 53 (26.5%), respectively. Overweight, sex, inadequate sleep, and stress were established as positive predictors of hypertension. The rising prevalence of hypertension and its modifiable risk factors in rural communities require prompt interventions directed at reversing these trends.

## 1. Introduction

In the past few decades, significant changes have occurred in the pattern of health and disease in many developing countries with noncommunicable diseases becoming a great contributor to the burden of disease [1]. The two major determinants of the increased burden are the epidemiological and demographic transitions [2]. The epidemiological transition has resulted in the replacement of previously predominant infectious disease pattern with chronic noncommunicable diseases as dominant public health problems. There is also a very complex interplay between infectious diseases and noncommunicable diseases as the latter are now linked to or are due to infectious causes [2]. In addition, with demographic transition, there is enlarging population of people assuming longer life span leading to a very rapid increase in the magnitude of non-communicable diseases. Furthermore, with changes in diet, a more sedentary life, use of tobacco products, alcohol consumption, and other drugs there is an increase in the risk of hypertension and other diseases associated with altered lifestyle [3].

Hypertension is a chronic noncommunicable condition of concern due to its role in the causation of coronary heart disease, stroke, and other vascular complications. The Nigerian National Expert Committee on noncommunicable diseases defined hypertension using a blood pressure (BP) cutoff of greater than or equal to 160/95 mmHg more than a decade ago [4]. However, recent classification defined hypertensive subjects as those with blood pressure greater than or equal to 140 mmHg systolic and 90 mmHg diastolic or those that self-reported the use of antihypertensive medication [5]. The BP is the single most useful test for identifying individuals at a risk of developing coronary heart disease [6]. Hypertension is the commonest cardiovascular disorder, posing a major public health challenge to a population in socioeconomic, demographic, and epidemiological transition [6]. In the 1950s to 1960s, hypertension was said to be rare in Africans, but in recent decades hypertension has become prevalent as high as 20% in adult Nigerians [7, 8]. A reasonable hypothesis is that more urban societies have a higher risk of hypertension when compared with the rural. Truly, previous studies had consistently reported a higher prevalence of hypertension in urban compared to rural areas agreeing with this position [8–10]. In an earlier study, the prevalence of hypertension (BP ≥ 160/95 mmHg) in a rural study in Nigeria was low, 5.9% [11]. Across the world, there is an increasing prevalence of hypertension with a projected prevalence rate of 29.2% by 2025 affecting about 1.54 billion individual globally [12].

The Nigerian National Expert Committee on noncommunicable diseases reported in 1997 prevalence rates of hypertension of 10–12% in urban setting and lower value, 8–10%, in rural population [4]. In 2003, the prevalence of hypertension in South West Nigeria was estimated to be 28.9% systolic in urban compared to 13.7% in rural and diastolic of 40.5% urban and 20.5% in rural areas, respectively [13]. A community based population based survey carried out in Abia State, South East Nigeria, in 2012 reported a general prevalence of hypertension of 31.8% and 30.0% systolic and 15% diastolic blood pressures [14]. However, more recent study showed increasing patterns in hypertension prevalence in rural areas [15]. A study carried out in a rural community in 2013 in Rivers State, Nigeria, reported a crude prevalence of 20.2% [16]. The apparently rising prevalence of hypertension in the rural communities may be attributed to inroads in lifestyle changes associated with “civilization” and the rapid “westernization” [16]. Some of these lifestyle changes serve as risk factors for hypertension.

A risk factor is defined as an attribute, characteristic, or exposure of an individual that is significantly associated with the development of a disease [6]. They are factors that make the occurrence of the disease more probable. Some of these risk factors are modifiable while others are immutable [6]. The nonmodifiable risk factors for hypertension are genetic factors, race, age, sex, family history, and personality. Hypertension on its own is a known independent and major risk factor for cardiovascular diseases and contributes greatly to the development of renal diseases, cardiac failures, and strokes [9, 11]. Hypertension is not only one of the risk factors for these diseases but also a condition with its own risk factors [6].

The modifiable risk factors are as a result of adoption of health-risky lifestyles such as cigarette smoking, high salt intake, consumption of saturated fat and dietary fiber, high alcohol consumption, physical inactivity, environmental stress, lower socioeconomic status, and some other factors [6, 17]. The modifiable factors are greatly affected by behavioral modifications and other interventions such as changing diet. This last factor requires further investigations.

Several studies have reported that these risk factors are significantly associated with the development of hypertension [14, 15, 17]. Cigarette smoking has been shown to increase BP. Smoking causes an acute increase in BP and heart rate and has been found to be associated with malignant hypertension [11]. The degree of risk of coronary heart diseases is directly proportional to the number of cigarette smoked per day [18]. There is evidence that the influence of smoking on coronary heart diseases is not independent of but also synergistic with other factors such as hypertension and elevated cholesterol. That means the effect is more additive. In the World Health Organization (WHO) report on global tobacco epidemic in 2008, Nigeria smoking prevalence was 17.1% and 0.9% in male and female adults, respectively [18]. Heavy consumption of alcohol is also positively associated with the occurrence of hypertension and it increases the risk of heart failure in persons with high BP [19].

Furthermore, the level of exposure of people to other risk factors of unhealthy diets, physical inactivity, undue stress and pressure, and harmful use of alcohol and other drugs has become higher in developing countries. This is contrary to the situation in high-income countries where comprehensive interventions at prompting healthier behavior, affordable and accessible health care services for early detection, effective treatment, and prevention of complications are in place [20]. Consequently, developing countries struggling with infectious diseases and hunger are also dealing with problems associated with diseases such as obesity [21].

In view of the rapid “westernization” of lifestyle in the rural African community with its associated lifestyle changes and in other to control the rising prevalence of hypertension there is the need to assess the distribution of the modifiable risk factors of hypertension in the rural areas with a view to devising suitable interventional strategies that will meet the peculiar nature of this population. The study was conducted to determine the burden of hypertension and its modifiable risk factors among two rural communities in Surulere Local Government Area (LGA) of Oyo State, South West Nigeria.

## 2. Materials and Methods

The study was a cross-sectional study carried out in purposively selected rural communities of Jabata and Ajase in Surulere LGA of Oyo State, South West Nigeria, between the months of February 2013 and March 2014. The LGA headquarter which is situated at Iresa Adu is about 129 km from Ibadan, the Oyo State capital, 21 km from Ladoke Akintola University of Technology Teaching Hospital (LTH), Ogbomoso, 10 km and 7 km to Jabata and Ajase, respectively. The LGA has about 117 communities spread within 10 Wards and an estimated population of 142,070 by 2006 national population census. The Department of Community Medicine, LTH Ogbomoso Model Rural Primary Health Care (PHC) Practicing Center, is situated in Jabata village. The two communities had similar characteristics and three major tribes, namely, Yoruba, Hausa/Fulani, and Igede. The paramount rulers of these communities are referred to Onijabata of Jabata and Alajase of Ajase, respectively. The majority of the population in the communities was involved in peasant farming. Many of the women were engaged in petty trading but this was mainly limited to selling of their farm products. Nonagricultural workers were mainly teachers in the primary and secondary schools and very few LGA staff and the LTH Ogbomoso-Jabata PHC personnel. Some inhabitants engage in crafts such as welding, carpentry, and molding of blocks.

The study population included all males and females of 18 years and above who were resident in the two communities at the time of the study and all were to participate in the study. The exclusion criteria included those who refused to participate and those who were unable to communicate effectively. The sample size was determined using the formula when prevalence of a disease is known; *N* = 4*pq*/*L* ^2^ where *N*, *p*, *q*, and *L* ^2^ were the sample size, prevalence, *p*, of hypertension from previous literature, 1 minus *p*, and permissible error in the estimate [15, 22]. At permissible error of 10% the calculated minimum sample size was 334. To allow for a nonresponse of 10%, three hundred and sixty-seven respondents comprising 166 male and 201 female were sampled.

A pretested interviewer-administered questionnaire adapted from WHO STEPS instrument for chronic disease risk factor surveillance was the tool for data collection [23]. The questionnaire was pretested in Gambari, a rural community similar in sociodemographic characteristics to Jabata and Ajase. The necessary amendments were made subsequently. All those who consented were interviewed. Information on sociodemographic data including age, gender, occupation, and educational status were collected. The questionnaire also elicited information on smoking habits, alcohol consumption, quantity of alcohol consumed, and average activity during work and leisure. History of prior knowledge of blood pressure status and family history of hypertension were also collected with the questionnaire. The research assistants were 600L medical students undergoing their community medicine posting at the Department of Community Medicine of the LTH, Ogbomoso, at the time of the study. They were trained on the techniques for data collection and questionnaire administration and offered practical demonstrations of blood pressure (BP) measurements and anthropometric indices which ensured standardization and uniformity in procedures using survey material recommended for noncommunicable disease [23]. Administration of the questionnaire was by face to face interview by the researcher and assistants. A quiet place at the survey site was chosen for the BP measurements. The researcher and resident doctors on rural practice posting at the LAUTECH PHC Practicing Center, Jabata, took the BP. The subjects BP were measured in sitting position between 7.00 am and 11.00 am on each day. After subjects had been comfortably seated they were allowed to rest for up to 10 minutes and without smoking and with the extended right arm held at the level of the heart; a cuff is applied to cover at least 40% of the right arm circumference in width; maximum inflation level was obtained from a standard mercury sphygmomanometer and auscultation was done over the brachial artery with the aid of a 15 cm stethoscope. The first appearance of the Korotkoff sounds as the cuff was deflated was taken as systolic and the disappearance of the sounds as diastolic, respectively. In cases where Korotkoff sounds remain audible despite deflation of the cuff, abrupt muffling of the sound was used for the diastolic measurement. The BP was measured to the nearest mmHg on two occasions at an interval of one minute. The systolic and diastolic pressures were measured three times over a period of 3 minutes and the lowest reading was recorded for each subject. For reasons of comparability, the data were recorded in a uniform way in all the outreaches. Hypertensive subjects were defined as those with blood pressure greater than or equal to 140 mmHg systolic and 90 mmHg diastolic or those that self-reported the use of antihypertensive medication [5].

Smokers were defined as those who had been smoking at least one cigarette per day, on the average, during the previous 30 days while nonsmokers were lifetime abstainers and occasional smokers [24].

Alcohol drinkers included individuals who took any amount of alcohol while nondrinkers included only abstainers. One unit of alcohol was defined as 1/2 bottle of beer or 1 glass of wine or 1 shot of gin/local gin/whisky. Alcohol consumption was classified as nondrinkers/abstainers; occasional drinkers as consumption of one unit/week; mild drinkers as consumption ≤14 units/week; moderate drinkers as consumption 15–21 units/week; and heavy drinkers as consumption >21 units/week [25, 26].

The Johnson Space Centre (JSC) physical activity scale was used to assess the participant activity level over the preceding three months [27, 28]. This 8-point Likert (0 to 7) tool consists of graded levels of activity ranging from 0 for subjects who avoid physical activities whenever possible to 7 for those who performed heavy physical activities regularly for more than 3 hours per week. The subjects were asked to select the appropriate score (0 to 7) which best described their general physical activity level. The subjects who selected a score of either 0 or 1 were classified as sedentary because these activity values represent either no physical activity or an insufficient and inconsistent amount of physical activity that was far below the minimum recommendations. Those subjects who selected a score of 2 or higher were classified as nonsedentary because these activity levels either approach or exceed the recommendations. The JSC physical activity scale has a strong independent relationship with maximal oxygen uptake in both sexes between 20 and 79 years of age group.

Job strain, social constraints, financial instability, and history of family distress were included subjectively under “stress” which significantly affects the daily life activities. Sleep adequacy was subjectively evaluated on the basis of sleep duration (7 to 8 hours), difficulty in initiating and maintaining sleep and early awakenings.

Anthropometric measurements were done with the standing heights of respondents measured with portable Leicester height stadiometer, graduated with subjects standing in an erect barefoot position, arms by side, and feet together with 0.1 cm precision. The body weights were measured with minimum clothing, barefooted, and without head coverings using a portable SECA Alpha (Model 770) electronic weighing scale, placed on firm horizontal surface. The scale was graduated to measure up to 0.1 kg with maximum weight of 150 kg. The weighing scale was standardized by reference to an individual of known weight who was weighed each day on a standard hospital based scale just before calibration. The performance of the instruments were checked on each day and recalibrated if found necessary.

The body mass index (BMI) was calculated using the standard formula weight in kg divided by the square of the height in meters (kg/m^2^). WHO classification of BMI was used in this study to grade BMI. Under weight—<18.5 2 2 Kg/m^2^; Normal Weight—18.5 to 24.9 Kg/m^2^; Overweight—25 to 29.9 Kg/m^2^; Obesity—>30 2 Kg/m^2^ [29].

Before the commencement of the study, ethical approval was obtained from the Research Ethics Review Committee of Ladoke Akintola University of Technology Teaching Hospital, Ogbomosho. The study complied with the Helsinki Declaration on research on human subjects of 1975 as revised in 1983. Permission to conduct this study was obtained from the traditional ruler of the two communities.

Verbal informed consent was also sought and obtained from each prospective participant after the purpose of the research was clearly and fully explained and understood by them. Furthermore, they were informed that participation was voluntary and participants had the right to refuse to participate or to withdraw from the research at anytime. Participants were informed of their BP readings and subjects found to be hypertensive were counseled and given referral letters to attend the LTH Ogbomoso for confirmation of hypertension and further management.

All data generated were revised, checked for completeness, and coded for computerized data entry. Data was analyzed using Statistical Package for Social Sciences version 17 software and the results presented descriptively as frequency and tables. Discrete variables were presented with use of tables and percentages. Fisher's exact and chi square test were used to test comparison of proportions and Student's *t*-test for normally distributed data. Multiple regression analysis was done to control potential confounding effect among the three sociodemographic variables of sex, age, and educational level. Pearson and Spearman's rho correlation test were used to determine the relationship between hypertension and its possible risk factors. Level of significance was set at *P* < 0.05.

## 3. Results

Three hundred and sixty-seven respondents comprising of 166 (45.2%) males and 201 (54.8%) females participated in this study. The age ranges were 18 to 83 for male and 18–87 years for female participants.

**Figure 1:**
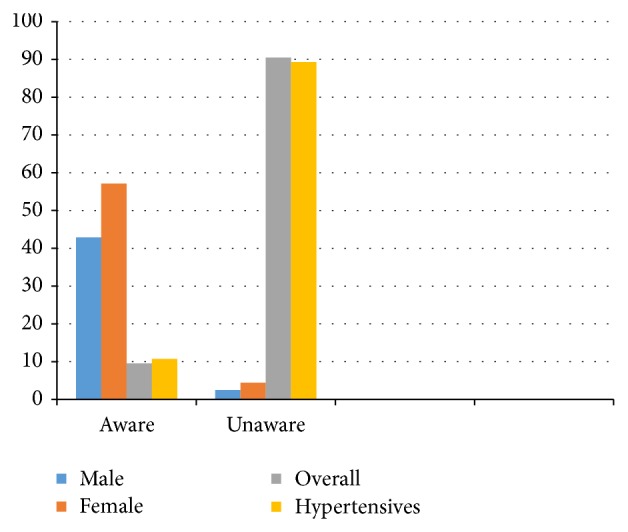
Awareness of Hypertension in subjects.

As shown in Figure 1, the overall awareness of all the subject was 9.5% with 57.14% in females and 42.86% in males. Among those found to be hypertensive, only 10.71% had awareness of their status while 89.29% were unaware.

Table 1 shows that the mean (SD) age of all subjects was 36.36 (±16.88) years while 35.80 (±16.40) and 36.93 (±17.28) years were for males and females, respectively. The mean systolic blood pressure was 124.00 (±16.93) mmHg (males 124.03 ± 17.53 and females 124.00 ± 15.99 mmHg; *P* > 0.05). No statistically significant gender difference was seen with respect to age, systolic and diastolic pressures, and BMI.

Table 2 shows the sociodemographic characteristics of the subjects with or without hypertension. All the participants were divided into two groups on the basis of their BP level as hypertensive with BP ≥ 140/90 mmHg, 89 (22.9%) and nonhypertensive with BP ≤ 140/90 mmHg 283 (77.1%). The prevalence of hypertension, in this study was 22.9% in the communities. Most of the subjects, 116 (31.6%), were in the 35 to 44 years age group and 220 (59.9.0%) were married while 153 (41.7%) had completed primary education. The male subjects had higher prevalence of hypertension, 49 (58.33%), than females 35 (41.67%). Hypertension predominates in subjects within the younger age groups 15–24 years (26.19%) and 25–34 years (25.00%) although the finding was not statistically significant. The prevalence of hypertension was higher in subjects without formal education compared to those with some years of education. These findings were statistically significant. The subjects occupation included farming 192 (52.3%), petty trading 70 (19.1%), government worker 19 (5.2%), and others 86 (23.4%). Prevalence of hypertension was higher, 45 (23.44%), in the farmers and the other occupational group, 24 (27.91%). These findings were not statistically significant.

Table 3 shows the modifiable risk factors for hypertension in the subjects with and without hypertension. Current smoking among hypertensive subjects was particularly low in this study; only 4 (4.76) of the smokers were found to be hypertensive; though this finding did not attain statistical significance. On the other hand, a higher proportion, 43 (51.2%), of the hypertensive subjects currently consumed alcohol. This finding was statistically significant, *P* < 0.05. Current consumption of alcohol was thus significantly associated with hypertension. Furthermore, hypertensive subjects had a higher prevalence of sleep inadequacy 67 (79.76%) than nonhypertensive subjects, 55 (35.10%). It was also observed that sedentary subjects had a slightly higher prevalence 25 (29.76%) of hypertension compared to those with active lifestyle, 66 (23.32%). Concerning stress, it was surprising that only about 5 (6.0%) of the subjects who had significant stress level were disposed to hypertension compared to 79 (94.0%) of those who insignificantly perceived stress. This finding was statistically significant, *P* < 0.05. In this study, it was observed that 25 (29.76%) subjects with abnormal weight had predisposition to hypertension, although 59 (70.23%) of normal weight subjects were also found to be hypertensive. These findings did not attain statistically significant value. In the female, only 13 (37.14%) of hypertensive subjects had used hormonal contraceptive compared to 22 (62.86%) of those who denied usage although the finding was not statistically significant.

Tables 4 and 5 show logistic regression analysis to assess the impact of sociodemographic and risk factors on the likelihood that subjects would develop hypertension.  Table 4 model contained seven independent variables (sex, age, educational level, occupation, marital status, job-related activities, and BMI) while Table 5 was on female subjects and contraceptives usage.

Although the full model was not statistically significant but the Hosmer and Lomeshow test support the model as being worthwhile with chi square of 3.72 and a significance level of .88 which is higher than 0.05. The significant value in the Hosmer-Lemeshow Test must be greater than 0.05 in well-fitted models. The model as a whole explained between 6.0% (Cox and Snell *R* square) and 9.2% (Nagelkerke *R* squared) of the variance in hypertensive status and correctly classified 77.7% of subjects. As shown in Table 4, only four of the independent variables made a unique statistically significant contribution to the model (sex, significant stress perception, inadequate sleep, and overweight). The odds for developing hypertension increased with increasing BMI. The association between BMI and hypertension increased further after adjusting other potential cofounders. Although the odds of having hypertension among smokers were 1.07 times that of nonsmokers, this finding was not statistically significant. The odds of having hypertension were almost the same in farmers and public servants but very low in traders after controlling potential cofounders. These findings did not attain statistical significance. The strongest predictor of hypertension was sleep inadequacy with odds ratio of 20.95; this indicated that subjects who had sleep inadequacy were over 20 times more likely to be hypertensive than those who had no adequate sleep, controlled for all other factors in the model. Conversely and quite surprisingly, the ratio of 0.29 for abnormal weight was less than 1, indicating that for every additional weight per subject, there were 0.32 times less likely to develop hypertension, controlled for other factors in the model. The likelihood of the use of contraceptives causing hypertension was not statistically significant (Table 5).

## 4. Discussion

The overall awareness of hypertension in the subjects was just 35 (9.5%) with 20 (57.14%) in females and 15 (42.86%) in males. Only 10.71% of the hypertensive subjects had awareness of their status. Studies in Africa have shown that many people with hypertension are unaware of their condition, many of those who are aware are not on treatment, and many of those treated are not well controlled [30]. This finding was not statistically significant. The reason for the poor awareness of hypertensive status might hitherto not be unconnected with the absence of well developed health care services in this rural communities and hence little or no health education (LTH Ogbomoso PHC practicing center is just about two years in existence). Previous studies also reported low level of awareness in most of Africa studies [8, 15, 19, 30]. Only 25 (29.7%) of those with previously known hypertension were receiving treatment. Effective treatment of hypertension could prevent 250,000 deaths each year in sub-Saharan Africa [31].

In this study, hypertension was found prevalent among 22.9% of the two rural communities surveyed in Surulere local government area of Oyo State, Nigeria.

Hypertension prevalence of 15.4% to 26.8% had been reported in previous studies [11, 30–34]. The prevalence of 22.9% in this study is lower than the 46.4% reported in rural community in Enugu State, South East Nigeria, and 26.8% in rural Mozambique [15, 35]. The higher prevalence in Enugu study may be because the study mainly involved middle age and the elderly when hypertension is more common. The 22.9% was, however, higher than the 15.2% in rural Ga District in Ghana, 20.2% in rural population in Rivers State Nigeria, 20.3% in rural West Africa, 17.3% in rural India, and 15.4% in rural communities in Maiduguri, Bornu State, North East Nigeria [9, 16, 30, 31, 33]. Earlier reports of one to two decades ago on rural prevalence of hypertension in sub-Saharan Africa gave a lower prevalence of less than 10.0%, a fact that was attributable to the use of higher cutoff value of 160/95 mmHg compared to the 140/90 mmHg used in this study [36]. The relatively high prevalence of hypertension in Surulere LGA of Oyo State could be attributed to transformation of the rural areas typified by the use of automobile transportation, mechanization of agriculture and of “modern” lifestyles with change in diet, and consumption of alcohol and smoking. Urban areas have generally reported higher prevalence from 6.4% to 41.0% although some of the disparities may be associated with factors of age, methods employed in BP measurement, and probably the cutoffs point for hypertension [7, 10].

Although there was statistically significant sex difference in hypertension prevalence in this study but the higher mean BP in males than females did not attain statistical significance. As documented in previous studies, the prevalence of hypertension in this study increased with age [15, 16, 32]. Hypertension prevalence was the highest, 50.00%, among subjects >75 years of age and the lowest, 16.28%, in those in 18–24 years age group. This finding was not statistically significant. Although the prevalence of hypertension was the highest, 25.24%, in subjects with no formal education and the lowest, 16.67%, in those with tertiary education, no statistically significant association was observed between hypertension prevalence and educational status. Having some degree of formal education carried a lower risk of developing hypertension compared to having no education at all [8]. Education influences health through promotion of active lifestyle behaviors.

A family history of hypertension is a significant risk factor in hypertension, obesity, and diabetes [34]. In this study, subjects with first-degree relative with family history of hypertension had statistically significant association with higher prevalence of hypertension compared to those with no family history, *P* < 0.05, a finding that is consistent with reports by previous researchers [36–38].

Alcohol consumption showed significant association with hypertension prevalence in our study. There were inconclusive evidences of the effect of alcohol on hypertension in most studies in Africa; some show association of regular and moderate alcohol intake and others show no association. Although it has been reported that regular and long time cigarette smoking is associated with higher blood pressure, this study did not show any statistically significant difference between smokers and nonsmokers (*P* = 0.61). Previous studies reported that regular and long cigarette smoking is associated with hypertension [16, 39]. Surprisingly, hypertension was diagnosed more frequently in sedentary subjects 25 (29.76%) than in physically active subjects 59 (70.24%) although the finding did not attain statistical significance.

Sleep inadequacy had significantly predisposed 67 (79.76%) of the subjects in this study to hypertension, *P* < 0.05. In a previous study depriving healthy subjects of sleep has been shown to acutely increase blood pressure and sympathetic nervous system activity [40]. Prolonged short sleep durations could also lead to hypertension through extended exposure to raised 24-hour blood pressure and heart rate, elevated sympathetic nervous system activity, and increased salt retention.

A statistically significant association was observed between stress perception by 5 (6.0%) of the subjects in this present study and hypertension, *P* < 0.05. Clearly defining stress is an important requirement for examining the relationship between stress and blood pressure. Stress has been defined as the internal state of an individual who perceives threats to his or her physical or psychic well-being. As such, an appropriate measure of stress should include some assessment of the individual's perception of each event or situation [41]. Exposure to chronic stress has been hypothesized as a risk factor for hypertension [42]. A statistically significant higher prevalence of hypertension among the overweight and obese subjects finding consistent with previous studies. The mean BMI of 21.99 (3.01) and 22.77 (3.77) for males and females, respectively, compares with those obtained for male and female in previous studies in rural areas locally and abroad [9–11, 16, 19, 43].

The logistic regression analysis established a positive and significant association with sex, perceived stress status, adequate/inadequate sleep, and overweight subjects. This is consistent with a previous study [16, 43]. Stress, inadequate sleep, overweight, and obesity are all known risk factors for hypertension.

This study was limited in that it was based on self-reporting of physical activity where the trend to overreport the actual level of physical activity is well known. Physical activity can be assessed subjectively using self-reported questionnaire or objectively (directly measured) using equipment such as pedometers or accelerometers. Self-reported questionnaires are commonly used in primary care centre because they are cheap and easy to use. However, both methods have drawbacks and are subjected to potential bias. Self-reported questionnaires may not be able to capture all types of physical activity, whereas certain devices may not be worn in activities such as swimming to measure physical activity. Previous literature recommended using both objective and subjective measurements to validate the results for better measurements and physical activity recording. The purposive sampling of the two rural communities could limit generalizability of the findings. Furthermore, since the study was based on self-reporting of the physical activity there could be trend to overreport the actual level of physical activity. Therefore, future longitudinal studies including multiple, repeated, precise measurements of the exposure and outcome from early age are more likely to be able to address the causality.

## 5. Conclusion

The risk factors associated with hypertension in our study are similar to previous reports, with a few exceptions. In this study, hypertension was associated with overweight, increasing age, sedentary lifestyles, sleep inadequacy, lack of education, and stress. The prevalence of hypertension is high (22.9%) in this rural communities but awareness was low, 10.71%. The apparently rising prevalence of hypertension in the rural communities may be attributed to inroads in lifestyle changes associated with “civilization” and the rapid “westernization” turning hypertension to a significant health problem in Nigerian rural communities. There is need for population-based strategies and deliberate health campaign targeted at regular screening and adequate management to prevent and reduce the incidence of hypertension. Rising distribution of sedentary lifestyles should be taken into account to identify at-risk groups and develop strategies to discourage this behavior. Elaborating the importance of adequate sleep and reduction of stressors with improvement in physical activities may make an important contribution to reduce the incidence of hypertension in the rural areas.

## Figures and Tables

**Table 1:** Characteristics of the subjects.

Variable	Female	Male	All subjects	*P* value
Mean age (years) (SD)	36.31 (15.65)	36.39 (17.65)	36.36 (16.88)	0.444
Mean BMI (SD)	21.99 (3.01)	22.77 (3.77)	22.47 (3.66)	0.337
Mean systolic BP (SD)	124.00 (15.99)	124.03 (17.53)	124.00 (16.93)	0.518
Mean diastolic BP (SD)	76.23 (11.22)	76.38 (12.26)	76.32 (11.85)	0.755

BP = blood pressure; BMI = body mass index.

**Table 2:** Sociodemographic characteristics of subjects with and without hypertension.

Variable	Hypertension		*χ* ^2^	*P* value
	Yes (84)	No (283)		
Age (years)				
15–24	7 (16.28)	36 (83.72)	9.82	0.13
25–34	5 (16.67)	25 (83.33)		
35–44	21 (18.10)	95 (81.90)		
45–54	22 (23.91)	70 (76.09)		
55–64	17 (31.48)	37 (68.52)		
65–74	10 (35.71)	18 (64.29)		
>75	2 (50.00)	2 (50.00)		
Sex				
Male	49 (29.52)	117 (70.48)	7.55	^*^0.01
Female	35 (17.42)	166 (82.59)		
Marital status				
Single	26 (21.49)	95 (78.51)	^**^0.23	0.90
Married	52 (23.64)	168 (76.36)		
Widow	6 (23.08)	20 (76.92)		
Educational status				
No formal education	26 (25.24)	77 (74.75)	^**^0.95	0.80
Primary	32 (20.91)	121 (79.08)		
Secondary	24 (24.24)	75 (75.75)		
Tertiary	2 (16.67)	10 (83.33)		
Occupation				
Farming	45 (23.44)	147 (76.56)	^**^3.33	0.34
Trading	11 (15.71)	59 (84.29)		
Government worker	4 (21.05)	15 (78.94)		
Others	24 (27.91)	62 (72.09)		

^**^Fisher's exact ^*^ *P* < 0.05.

**Table 3:** Modifiable risk factors in hypertensive and nonhypertensive subjects.

Variable	Hypertension		*χ* ^2^	*P* value
	Yes (%)	No (%)		
Family history				
Yes	24 (28.57)	11 (3.90)	45.7	^*^0.00
No	60 (71.43)	272 (96.11)		
Current smoking				
Yes	4 (4.76)	10 (3.53)	0.27	0.61
No	80 (95.2)	273 (96.50)		
Alcohol consumption				
Yes	43 (51.20)	106 (37.50)	5.07	^*^0.02
No	41 (48.80)	177 (62.54)		
BMI				
Normal	59 (70.23)	220 (77.74)	^**^3.02	0.23
Abnormal	25 (29.76)	63 (22.26)		
Physical activity				
Sedentary	25 (29.76)	66 (23.32)	1.44	0.23
Nonsedentary	59 (70.24)	217 (76.68)		
Perceived stress				
Significant	5 (6.00)	60 (21.20)	10.34	^*^0.01
Insignificant	79 (94.00)	223 (78.80)		
Sleep				
Adequate	17 (20.24)	228 (64.90)	106.23	^*^0.00
Inadequate	67 (79.76)	55 (35.10)		
^ #^Contraceptive usage				
Yes	13 (37.14)	40 (24.00)	2.53	0.11
No	22 (62.86)	126 (76.00)		

^**^Fishers exact ^*^ *P* < 0.05.

^#^In female subjects only.

**Table 4:** Logistic regression model predicting likelihood of modifiable risk factors for hypertension.

Variable	*B*	SE	Wald	df	*P*	OR	95% CI for OR	
							Lower	Upper
Sex	−1.13	0.45	6.32	1	^*^0.01	0.32	0.13	0.78
Age								
15–24	−0.61	0.43	2.02	1	0.16	0.54	0.24	1.26
25–34	0.08	0.62	0.02	1	0.10	1.08	0.32	3.68
35–44	0.35	0.50	0.50	1	0.48	1.42	0.54	3.75
45–54	−0.62	0.70	0.80	1	0.37	0.54	0.14	2.09
55–64	0.29	0.63	0.21	1	0.65	1.33	0.39	4.60
65–74	1.07	1.65	0.43	1	0.51	2.94	0.12	73.77
Educational level								
None	−0.00	0.42	0.00	1	0.99	0.10	0.44	2.27
Primary	0.41	0.52	0.63	1	0.43	1.51	0.55	4.14
Secondary	0.56	1.06	0.28	1	0.60	1.76	0.22	13.95
Occupation								
Public servant	−0.12	0.85	0.02	1	10.88	0.88	0.17	4.65
Trading	0.12	0.77	0.03	1	0.87	1.13	0.25	5.12
Farming	1.06	0.84	1.60	1	10.21	2.88	0.56	14.89
Marital status								
Single	0.54	0.44	1.50	1	10.22	1.71	0.72	4.06
Married	1.03	0.79	1.73	1	0.19	2.81	0.60	13.10
Smoking	0.07	0.90	0.01	1	0.94	1.07	0.18	6.19
Alcohol	−0.63	0.36	3.06	1	0.08	0.53	0.26	1.08
Stress	−1.96	0.67	8.61	1	^*^0.00	7.10	1.92	26.25
Sleep	3.04	0.36	70.03	1	^*^0.00	20.95	10.27	42.72
Job-related physical activity								
Sedentary	−0.50	0.38	1.67	1	0.20	0.61	0.29	1.29
Vigorous	−0.06	0.56	0.01	1	0.91	0.94	0.32	2.80
BMI								
Normal	−2.08	0.62	11.31	1	^*^0.00	0.13	0.04	0.42
Abnormal	−1.25	0.63	3.90	1	^*^0.49	0.29	0.08	1.00
Constant	−3.99	1.32	6.62	1	0.01	0.03		

^*^ *P* < 0.05; OR = odds ratio; CI = confidence interval.

**Table 5:** Logistic regression model predicting likelihood of hormonal contraceptives as a cause of hypertension among female subjects.

Variable	*B*	SE	Wald	df	*P*	OR	95% CI for OR	
							Lower	Upper
Contraceptive use	0.62	0.39	2.49	1	0.12	1.86	0.86	4.03
	−1.75	0.23	57.05	1	^*^0.00	0.18	0.18	

^*^ *P* < 0.05; OR = odds ratio; CI = confidence interval.
